# Kidney effects of Glucagon-Like Peptide 1 (GLP1): from molecular foundations to a pharmacophysiological perspective

**DOI:** 10.1590/2175-8239-JBN-2024-0101en

**Published:** 2024-11-08

**Authors:** Jorge Rico-Fontalvo, Maricely Reina, María José Soler, Mario Unigarro-Palacios, Juan Pablo Castañeda-González, Javier Jiménez Quintero, María Raad-Sarabia, Thyago Proença de Moraes, Rodrigo Daza-Arnedo

**Affiliations:** 1 Asociación Colombiana de Nefrología e HTA, Comité de Riñón, Diabetes y Metabolismo, Bogotá, Colombia.; 2 Universidad Simón Bolívar, Facultad de Ciencias de la Salud, Departamento de Nefrología, Barranquilla, Colombia.; 3 Fundación Universitaria de Ciencias de la Salud, Hospital San José, Departamento de Nefrología, Bogotá, Colombia.; 4 Hospital Universitario Vall de Hebron, Servicio de Nefrología, Barcelona, España.; 5 Nephrology and Transplantation Research Group, Vall d’Hebron Institut de Recerca (VHIR), Nephrology Department, Vall d’Hebron Hospital Universitari, Vall d’Hebron Barcelona Hospital Campus, Barcelona, Spain. Centro de Referencia en Enfermedad Glomerular Compleja del Sistema Nacional de Salud de España (CSUR), Barcelona, España.; 6 Redes de Investigación Cooperativa Orientadas a Resultados en Salud (RICORS), Instituto de Salud Carlos III (RD21/0005/0031), Spain.; 7 Fundación Universitaria de Ciencias de la Salud, Hospital San José, Departamento de Endocrinología, Bogotá, Colombia.; 8 Fundación Universitaria de Ciencias de la Salud, Instituto de Investigaciones, Bogotá, Colombia.; 9 Universidad Militar Nueva Granada, Bogotá, Colombia.; 10 Universidad del Sinú, Departamento de Medicina Interna, Cartagena, Colombia.; 11 Pontificia Universidade de Catolica do Parana (PUCPR), Curitiba, PR, Brazil.

**Keywords:** Albuminuria, Diabetes, Incretins, Obesity, Kidney disease

## Abstract

GLP1 receptor agonists (GLP1-RAs) are drugs that mimic the effects of the incretin hormone GLP1 and were initially introduced in medicine for the treatment of diabetes in 2005 and for obesity in 2014. Over time, data from secondary and exploratory objectives of large randomized controlled-trials suggested that GLP1-RAs could also exert renal action by slowing the progression of kidney disease in patients with and without diabetes. Based on this rationale, the Flow study (1 mg semaglutide vs placebo) was designed and recruitment began in 2019 until May 2021. The recently published results confirmed the effect of semaglutide in reducing the composite renal outcome. However, similar to SGLT2 inhibitors, the potential mechanisms behind the renal effects of GLP1-RAs still need to be elucidated. The aim of this review is to address the different physiological mechanisms of GLP1-RAs at the renal level, using evidence from experimental studies and current scientific literature.

## GLP1 Production and Secretion

Glucagon-Like Peptide 1 (GLP1) is a polypeptide composed of 30 amino acids that originates from post-translational processes of the proglucagon gene. Its formation requires amidation at the carboxyl end, facilitated by a peptide-convertase enzyme complex^1,2^. These processes of translation, amidation, and release primarily occur in L-type cells located in the gastrointestinal epithelium, particularly in the ileum and colon^3^. Amidation is crucial for GLP1, as it impacts metabolic and endocrine functions, including the regulation of glucose, lipids, and gastric emptying^4^. In the 1980s, during studies on proglucagon genes, GLP1 was discovered and characterized. Initially considered inactive, further research revealed that post-translational amidation was essential for its activation. It was determined that the active and functional forms in the body are GLP1(7-37) and GLP1(7-36)-amide, detectable in the peripheral circulation^2,5^.

## Physiological Effects of GLP1

GLP1 performs a variety of physiological functions that focus on glucose homeostasis, depending on the glycemic and nutritional status of patients^6,7^. In fasting conditions, the average concentration of GLP1(7-36)-amide is 7.6 ± 1 pM. However, 90 minutes after eating, there is a significant increase, reaching 41.6 ± 5 pM. In contrast, GLP1(7-37) shows a more modest increase after eating, with concentrations of 6.6 ± 1 pM while fasting and 10 ± 1 pM after 90 minutes from food intake^5^.

The GLP1 receptor (GLP1R) consists of 463 amino acids and includes eight hydrophobic domains. The key region of the GLP1(7-36)-amide peptide, responsible for its binding to the receptor, is located in amino acids 7 to 21. Notably, the amino acid at position 7 (histidine) at the N-terminal end of GLP1 features an imidazole side chain and a free amino group. The imidazole side chain is crucial for the interaction with the GLP1R, while the free amino group is the site where the enzyme dipeptidyl peptidase-4 (DPP-4) carries out enzymatic inactivation^2,8,9,10^.

GLP1 exerts its effects by activating receptors belonging to the family of metabotropic G protein-coupled receptors, which are distributed across various organs such as the pancreas, lungs, heart, kidneys, stomach, intestine, pituitary gland, vagus nerve, and multiple regions of the central nervous system^11^.

GLP1 acts on the pancreas with the purpose of reducing blood glucose levels. This is achieved by increasing the synthesis and release of insulin, as well as stimulating the proliferation and inhibiting apoptosis of beta cells as demonstrated in animal and in vitro studies^7,11,12,13^. It is interesting to note that this increase in insulin secretion is proportional to blood glucose levels, and this is one of the factors that likely contributes significantly to the low incidence of hypoglycemia with the use of GLP1-RAs.

## Pharmacokinetics and Pharmacodynamics of GLP1 Receptor Agonists

GLP1-RAs are a group of drugs that act as hypoglycemic agents by mimicking the action of the endogenous GLP1 peptide. They activate the GLP1R, triggering glucose-dependent insulin secretion, inhibiting glucagon release, and delaying gastric emptying. Additionally, these drugs have a longer half-life than endogenous peptides because their structure makes them resistant to inactivation by the DPP-4 enzyme 4^13^.

Currently, there are six approved drugs in this category: exenatide, liraglutide, semaglutide, albiglutide, lixisenatide, and dulaglutide. However, albiglutide is not commercially available. Each of these drugs reduces glycated hemoglobin levels by approximately 1%, with a greater impact on patients with poorer glycemic control^14^.

In addition to the structural differences between GLP1 agonists and endogenous GLP1 peptide, GLP1RAs are structurally different from each other, giving them unique pharmacokinetic and pharmacodynamic characteristics. According to their structure, they can be divided into those of human origin, those synthesized from modifications of active fragments of physiological GLP1, and those generated by replicating the structure of exendin-4, a natural 39-amino acid peptide naturally resistant to the action of the DPP-4 enzyme^15^. Classification according to pharmacokinetic properties divides these drugs into two different groups: short-acting agonists (exenatide and lixisenatide) with a half-life of approximately 2 to 3 hours and long-acting agonists (dulaglutide, liraglutide, semaglutide, albiglutide, and long-acting exenatide) with a half-life of 7 to 13 days^16^.

## Kidney Effects of GLP1-RAs

GLP1R has been identified in different parts of the tubuloglomerular unit. Human studies have identified its expression in proximal tubular cells and in preglomerular vascular smooth muscle cells^17^ whilst in animal models, various studies have localized GLP1R in glomerular cells and the proximal portion of the proximal convoluted tubule^18,19^.

Additionally, GLP1-RAs seem to induce an increase in sodium excretion and urinary volume through direct action on the proximal tubule. This effect, which was dose-dependent, was also associated with a reduction in GFR, suggesting a potential renoprotective effect of GLP1-RAs since the mid-2000s^20,21^.

Most of the evidence on hemodynamic impacts at the glomerular level comes from animal studies and in vitro models, which somewhat limits the extrapolation of these findings to physiological conditions in humans^22^.

Some animal models have provided evidence suggesting that GLP1 is a physiologically relevant natriuretic factor that contributes to sodium balance, partly through the tonal modulation of the activity of sodium-hydrogen exchanger isoform 3 (NHE3) in the brush border of the proximal convoluted tubule^23,24^. The natriuresis induced by these hormones activates tubuloglomerular feedback and, as a result, promotes reflex vasoconstriction of the glomerular afferent arteriole^19^.

On the other hand, the vascular role of GLP1-RAs seems to be explained by their action on endothelin-1, a potent vasoconstrictor that can contribute to renal dysfunction and the progression of clinical disease^25^. In vitro and in vivo studies have demonstrated the action of GLP1-RAs through the suppression of endothelin-1 at the vascular level by decreasing the phosphorylation of nuclear factor kappa B (NF-κB) in the presence of elevated glucose concentrations, as occurs in patients with type 2 diabetes mellitus (DM2). This reduces vasoconstriction and improves renal blood flow^26^.

**Figure 1:**
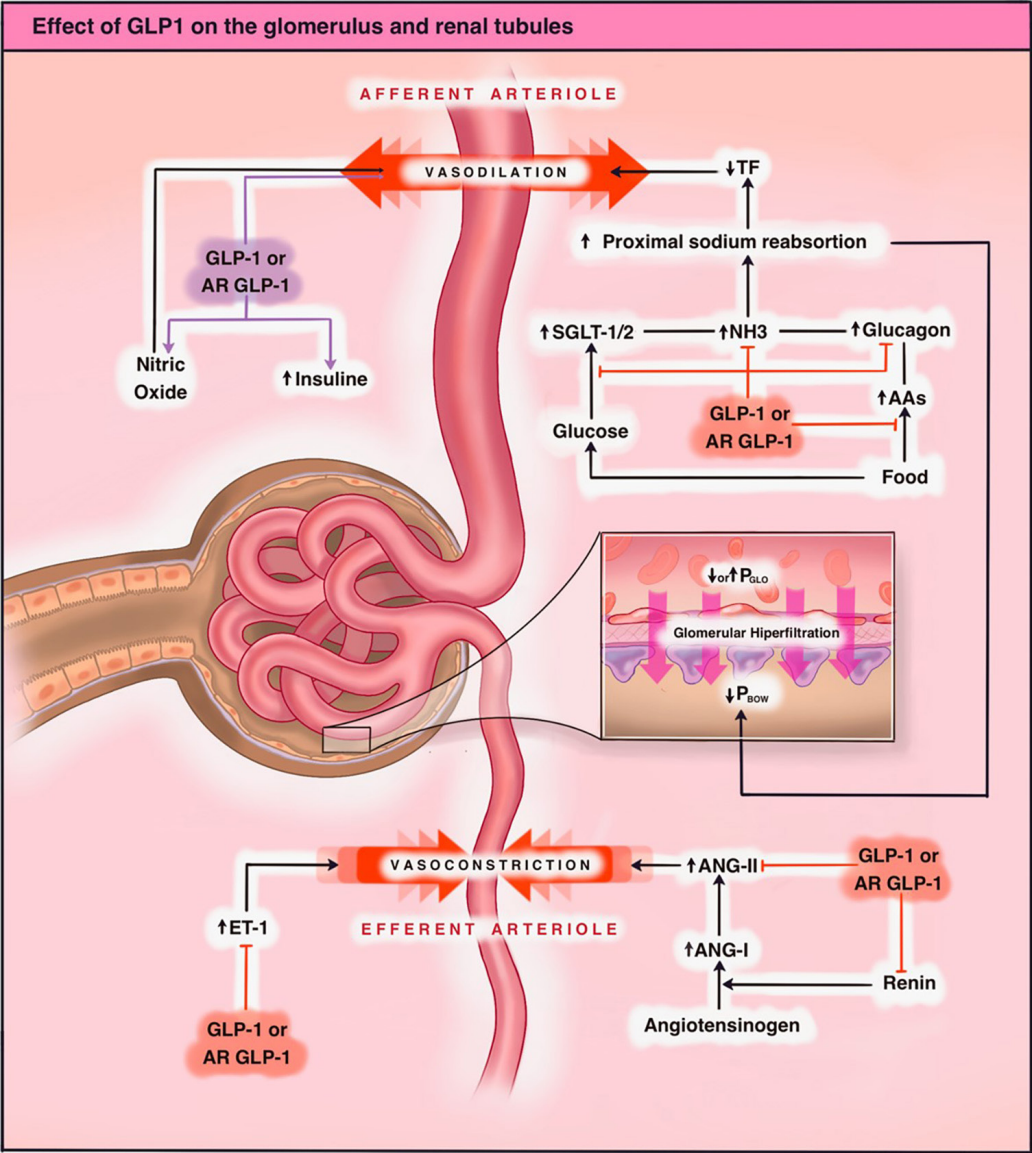
Effect of GLP1 on the glomerulus and renal tubules: Stimulation of the GLP1 receptor induces vasodilation of the afferent arteriole mediated by an increase in nitric oxide availability (NO-mediated vasodilation). There is also direct vasodilation on the afferent arteriole. This results in an increase in intraglomerular pressure. On the other hand, GLP1 decreases vascular resistance in the efferent arteriole through antagonizing mediators such as endothelin 1 (ET1) and angiotensin 2 (ANGII), resulting in postglomerular vasodilation and consequently a decrease in intraglomerular pressure. In the tubules, GLP1 induces a natriuretic effect, decreasing the activity and expression of sodium-glucose cotransporters type I and II (SGLT1/2) as well as reducing the activity of the NH3 exchanger through phosphorylation, leading to increased Na+ presence in the macula densa, restoring tubuloglomerular feedback with afferent vasoconstriction and reduction of intraglomerular pressures. There is also a decrease in glucagon release, which is related to reduced Na+ reabsorption in the proximal convoluted tubule. In summary, the net effect on glomerular filtration rate will depend on the magnitude of the dominant effect on mechanisms with opposing effects, the baseline glomerular filtration rate, and the individual characteristics of each patient. GLP1: Glucagon-like peptide-1.

In addition, tubuloglomerular feedback activation reduces renin secretion through an inhibitory signal in the juxtaglomerular apparatus, reflexively inducing vasodilation of the glomerular efferent arteriole. In this way, GLP1 is part of the regulatory mechanisms involved in the homeostasis of the renin-angiotensin-aldosterone system, which is commonly compromised in patients with nephropathy^27,28^. Additional research has reported that GLP1 may downregulate tissue expression of angiotensin II, which could lead to beneficial physiological processes by reducing glomerular hypertension, aldosterone secretion, and tone of the renal efferent arterioles^28^. In this way, various antagonistic mechanisms are evidenced that paradoxically modify renal hemodynamics; however, there are also other mechanisms that tip the balance toward a net nephroprotective effect in patients using analogs of these medications. Figure 1 summarizes the known glomerular and tubular renal effects of GLP1.

Within these additional mechanisms, related research has revealed that GLP1-RAs decreases the expression of AT1 receptors, favoring the function of AT2 receptors in glomerular capillaries and the proximal tubules of the nephron. This effect contributes to the reduction of angiotensin 2-induced fibrosis^28^. On the other hand, some animal models have established that GLP1-RAs administration increases the activity of angiotensin-converting enzyme in different organs, particularly in the kidney, whose enzymatic activity is compromised in scenarios of renal failure^29^.

**Figure 2:**
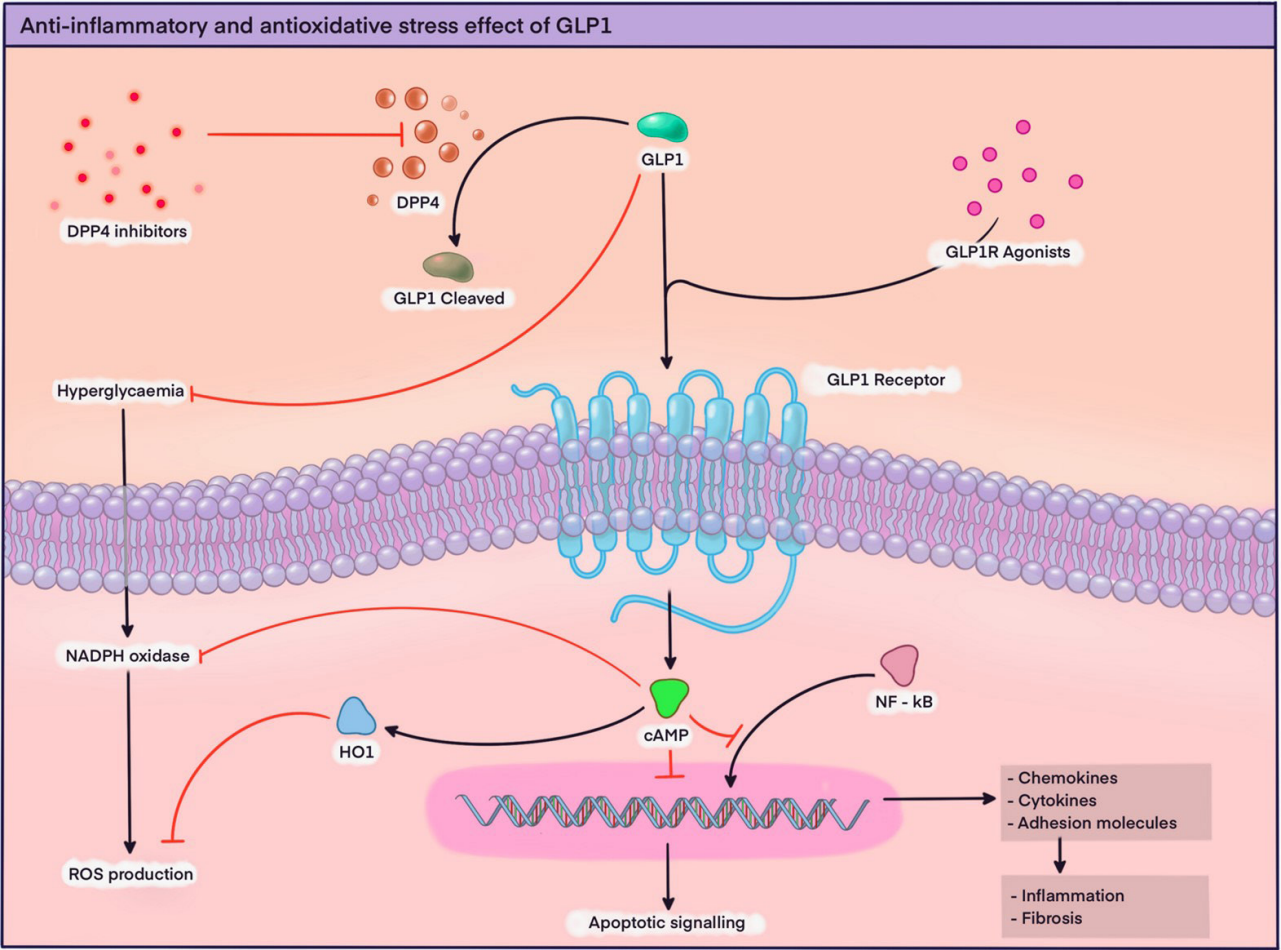
Anti-inflammatory and antioxidative stress effect of GLP1: Stimulation of the GLP1 receptor activates intracellular signaling pathways such as protein kinase A, leading to a subsequent increase in the concentration of second messengers such as cyclic adenosine monophosphate (cAMP), which exerts an inhibitory effect on nicotinamide adenine dinucleotide phosphate (NADPH) oxidase, an important enzyme in the production of reactive oxygen species (ROS). Additionally, there is a positive regulation of heme oxygenase 1 (HO1), which inhibits ROS production. The increase in cAMP concentrations negatively regulates apoptotic signaling and the activity of the nuclear factor Kappa Beta (NF-κB), resulting in a subsequent decrease in the production of proinflammatory cytokines and chemokines. DPP4: Dipeptidyl peptidase-4, GLP1: Glucagon-like peptide-1.

Another potential nephroprotective effect of GLP1 is related to its antioxidant function, which is carried out through the downregulation of nuclear factor-kB mediated by superoxide and the suppression of the NADPH oxidase 4 (NOX-4) enzyme^30,31^. This enzyme is inducible through an angiotensin 2-dependent transduction cascade. NOX- 4 promotes the production of reactive oxygen species, leading to hyperactivation of the renin-angiotensin-aldosterone system, as well as endothelial dysfunction and diabetic nephropathy. In addition to inhibiting tissue production of angiotensin 2, GLP1 activates the AMPc-dependent transduction pathway and protein kinase A, which negatively counteracts NOX-4 expression and reduces oxidative stress-derived effects^32,33^. Figure 2 describes the anti-inflammatory mechanisms and reduction of oxidative stress induced by GLP1.

The antialbuminuric effect related to the physiological functions of GLP1 has been reported as an underlying phenomenon that is associated with the decrease in plasma renin activity, induction of natriuresis, and reduction of oxidative stress^21^.

## Kidney Outcomes

With the regulatory changes instituted by the FDA at the end of the 2000s, large cardiovascular safety studies became mandatory for new drugs to treat diabetes. This requirement provided the opportunity to explore relevant secondary outcomes, including those related to kidney disease, which are of particular interest to us.

Over the past several years, various studies on different GLP1-RAs have shown promising data regarding this new class of medications for the treatment of chronic kidney disease (CKD) progression. The LEADER study^34^, found that the administration of liraglutide in diabetic patients was associated with a significant reduction in the incidence of nephropathy as a microvascular event compared to the placebo group (HR 0.78; 95% CI 0.67–0.92; p = 0.003). Additionally, the use of liraglutide was also linked to a lower incidence of chronic kidney disease, highlighting a significant preservation in the number of events with a glomerular filtration rate (GFR) between 30 and 59 mL/min/1.73 m^2^.

Similarly, data from the SUSTAIN-6 study^35^, which compared the administration of semaglutide with placebo, revealed a notable nephroprotective function of GLP1-RAs. This was associated with a significant reduction in the incidence of macroalbuminuria and a lower number of GFR reduction events. Furthermore, the REWIND study^36^ revealed significant differences favoring the use of dulaglutide compared to placebo regarding de novo macroalbuminuria and reduction of renal function with GFR > 30 mL/min/1.73 m^2^. The HARMONY study reported that in patients treated with albiglutide, there are a lower incidence of renal failure with GFR ≥60 to <90 mL/min/1.73 m^2^ compared to the placebo group^37^. In the AMPLITUDE-O study^38^, a lower incidence of macroalbuminuria and deterioration was found in renal function in diabetic patients treated with efpeglenatide compared to those who received placebo. This underscores the need for further evidence to confirm whether these medications are truly nephroprotective in patients with CKD associated with type 2 diabetes. Real-world studies have confirmed the same results found in controlled trials, demonstrating the efficacy, safety, and reduction of albuminuria with GLP1-RAs^39,40^.

Given all these promising results, it is only natural to design and conduct a study with GLP1-RAs focusing primarily on kidney outcomes. The FLOW study with semaglutide was the first study specifically designed to confirm the renal benefits of this medication in a population of diabetic patients at very high cardiovascular risk^41^. The use of 1 mg of semaglutide resulted in a 24% reduction in the primary composite outcome, which included a persistent reduction in eGFR of more than 50%, reaching an eGFR of less than 15 mL/min/1.73 m^2^, initiation of renal replacement therapy, and renal or cardiovascular death. All the results of the other pre-specified outcomes in the hierarchical analysis were also in favor of semaglutide, including the comparison of the slopes of the glomerular filtration rate (GFR) curves, major adverse cardiovascular events (MACE), and even all-cause mortality.

**Figure 3:**
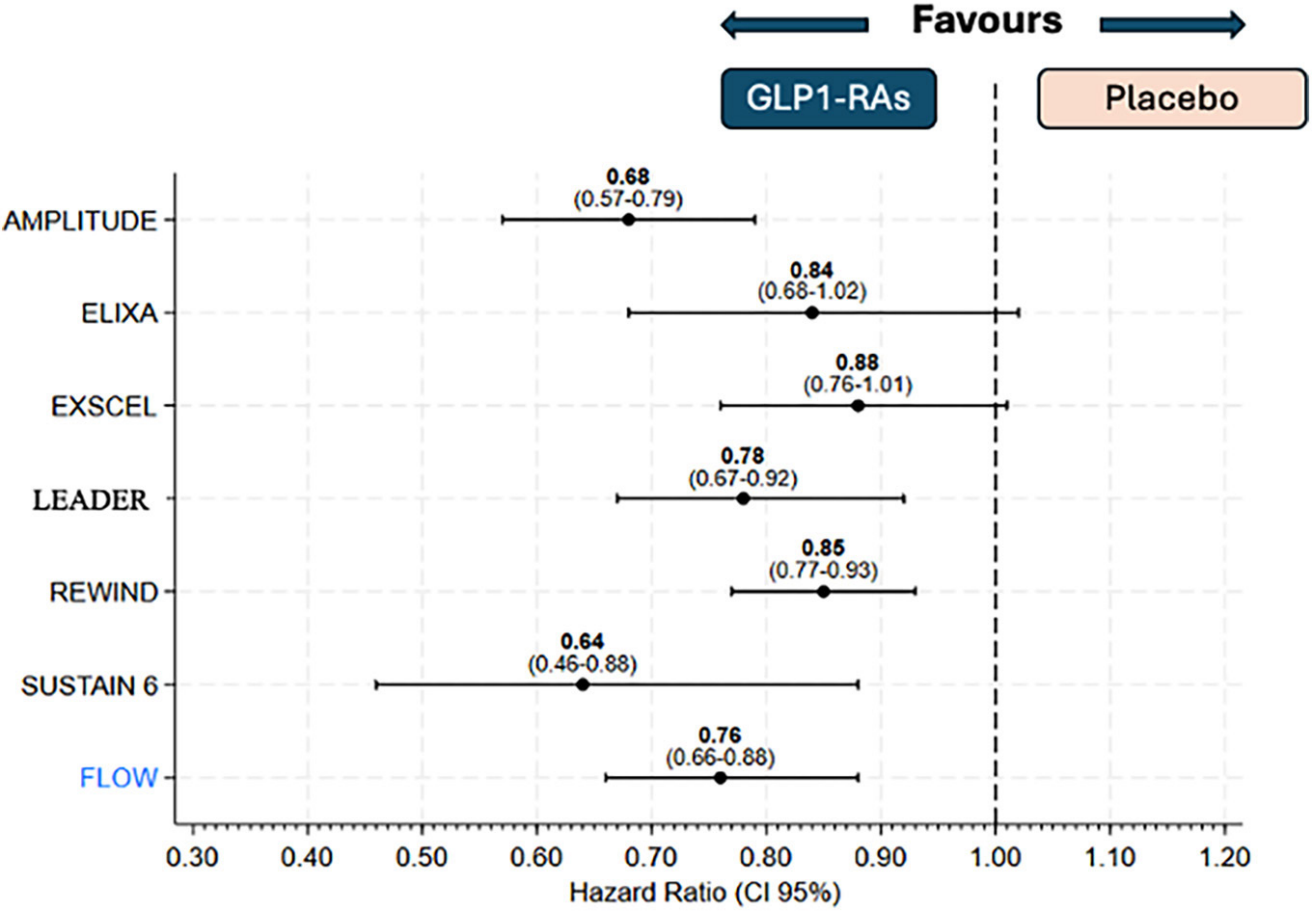
Composite kidney outcomes in GLP1 clinical trials.

The publication of the FLOW study, which occurred no more than two weeks before the conclusion of this review, has not yet provided a mediation analysis. This analysis would help determine how much of the observed effect was due to indirect effects, such as weight loss and HbA1c reduction, as opposed to a potential direct effect of semaglutide. The latter can include better control of oxidative stress, an anti-inflammatory action, induction of natriuresis, and a reduction of glomerular hypertension. Figure 3 summarizes the individual results of all these studies for a better visualization of the impact of GLP1-RAs on renal outcomes.

## Usage of GLP1 Analogues in Non-Diabetic Obese Patients

Because of the good results with GLP1-RAs in diabetic patients, it was to be expected that the study of the effects of these drugs would also begin to be explored in patients without diabetes. This is similar to what we have recently observed in nephrology with SGLT2 inhibitors and is currently ongoing with finerenone.

Data from over forty clinical trials conducted in obese patients without diabetes indicate an absolute nephroprotective effect^42,43,44^. In the STEP 3 trial, a significantly higher percentage of patients with normal renal function (eGFR ≥90 mL/min/1.73 m^2^) was observed in the semaglutide-treated group compared to the placebo group (68.8% vs. 65.2%)^45^. Conversely, in a double-blind randomized clinical trial, no cases of renal deterioration or failure were observed in patients treated with exenatide and dapagliflozin. However, it is important to note that these events occurred in all arms of the study, underscoring the need for continued monitoring^46^. Recently, the TRIAL study demonstrated a lower incidence of death from renal causes, initiation of long-term renal replacement therapy (dialysis or transplant), persistent eGFR below 15 mL per minute per 1.73 m^2^, persistent 50% reduction in eGFR compared to baseline, and initiation of persistent macroalbuminuria in patients treated with semaglutide compared to placebo^47^. Additionally, in the exploratory study by Heerspink et al^48^, data from the STEP 1, STEP 2, and STEP 3 trials were analyzed, finding that the urine albumin-to-creatinine ratio improved in a greater proportion of patients taking semaglutide 1.0 mg and 2.4 mg compared to placebo. It is also noteworthy that a significant decrease in albuminuria was observed in the non-diabetic population involved in these studies. This suggests the possibility of direct nephroprotective effects of the medication in this group, in addition to the indirect effect of weight reduction.

Finally, we recently published the results of the SELECT trial on kidney outcomes. Using data from over 16,000 patients, half of whom received semaglutide at a dose of 2.4 mg weekly, the authors reported an improvement in renal outcomes even in patients without diabetes. The composite outcome of the SELECT study differs from that of the FLOW study in that it includes the onset of persistent macroalbuminuria as one of its components. The observed reduction in the composite outcome was very similar to that of the FLOW study, with a 22% reduction over 104 weeks of follow-up^49^.

## Conclusions

Every day we learn more about the renal effects of GLP1-RAs. These mechanisms of action in the renal system can be classified into direct and indirect. However, we must continue to gain a better understanding of the molecular pathways through which GLP1-RAs exert their impact on renal function, as clinical data suggest that this therapeutic class of drugs could have significant nephroprotective effects. Most of the literature providing clinical data on renal outcomes is derived from pivotal studies in diabetic patients with or without obesity. Therefore, we must both improve the evidence in obese patients without diabetes and conduct specific studies in populations with already defined chronic kidney disease.
