# Supplementary material for: Kidney effects of Glucagon-Like Peptide 1 (GLP1): from molecular foundations to a pharmacophysiological perspective
Source: J Bras Nefrol. 2024 Nov 8;46(4):e20240101. doi: 10.1590/2175-8239-JBN-2024-0101en (PMC11548866; doi:10.1590/2175-8239-JBN-2024-0101en)
Supplement: Translation

# Translation: Efeitos renais do Peptídeo 1 Semelhante ao Glucagon (GLP1): das bases moleculares a uma perspectiva farmacofisiológica

**DOI:** 10.1590/2175-8239-JBN-2024-0101pt

**Authors:** Jorge Rico-Fontalvo, Maricely Reina, María José Soler, Mario Unigarro-Palacios, Juan Pablo Castañeda-González, Javier Jiménez Quintero, María Raad-Sarabia, Thyago Proença de Moraes, Rodrigo Daza-Arnedo

**Affiliations:** 1 Asociación Colombiana de Nefrología e HTA, Comité de Riñón, Diabetes y Metabolismo, Bogotá, Colômbia.; 2 Universidad Simón Bolívar, Facultad de Ciencias de la Salud, Departamento de Nefrología, Barranquilla, Colômbia.; 3 Fundación Universitaria de Ciencias de la Salud, Hospital San José, Departamento de Nefrología, Bogotá, Colômbia.; 4 Hospital Universitario Vall de Hebron, Servicio de Nefrología, Barcelona, Espanha.; 5 Nephrology and Transplantation Research Group, Vall d’Hebron Institut de Recerca (VHIR), Nephrology Department, Vall d’Hebron Hospital Universitari, Vall d’Hebron Barcelona Hospital Campus, Barcelona, Spain. Centro de Referencia en Enfermedad Glomerular Compleja del Sistema Nacional de Salud de España (CSUR), Barcelona, Espanha.; 6 Redes de Investigación Cooperativa Orientadas a Resultados en Salud (RICORS), Instituto de Salud Carlos III (RD21/0005/0031), Espanha.; 7 Fundación Universitaria de Ciencias de la Salud, Hospital San José, Departamento de Endocrinología, Bogotá, Colômbia.; 8 Fundación Universitaria de Ciencias de la Salud, Instituto de Investigaciones, Bogotá, Colômbia.; 9 Universidad Militar Nueva Granada, Bogotá, Colômbia.; 10 Universidad del Sinú, Departamento de Medicina Interna, Cartagena, Colômbia.; 11 Pontificia Universidade de Catolica do Parana (PUCPR), Curitiba, PR, Brasil.

**Keywords:** Albuminúria, Diabetes, Incretinas, Obesidade, Doença Renal

## Abstract

Os agonistas do receptor de GLP1 (GLP1-RAs) são medicamentos que imitam os efeitos do hormônio incretínico GLP1. Eles foram inicialmente introduzidos na medicina para o tratamento do diabetes em 2005 e para a obesidade em 2014. Com o passar do tempo, dados provenientes de objetivos secundários e exploratórios de amplos ensaios clínicos randomizados sugeriram que os GLP1-RAs também poderiam exercer ação renal ao retardar a progressão da doença renal em pacientes com e sem diabetes. Com base nesse raciocínio, o estudo Flow (semaglutida 1 mg *vs.* placebo) foi desenhado e o recrutamento começou em 2019, estendendo-se até maio de 2021. Os resultados publicados recentemente confirmaram o efeito da semaglutida na redução do desfecho renal composto. No entanto, assim como os inibidores do SGLT2, os mecanismos potenciais por trás dos efeitos renais dos GLP1-RAs ainda precisam ser elucidados. O objetivo desta revisão é abordar os diferentes mecanismos fisiológicos dos GLP1-RAs em nível renal, utilizando evidências de estudos experimentais e da literatura científica atual.

## Produção e Secreção de GLP1

O peptídeo 1 semelhante ao glucagon (GLP1) é um polipeptídeo composto por 30 aminoácidos, originado de processos pós-traducionais do gene do proglucagon. Sua formação requer amidação na extremidade carboxila, facilitada por um complexo enzimático peptídeo-convertase^1,2^. Esses processos de tradução, amidação e liberação ocorrem principalmente em células tipo L localizadas no epitélio gastrointestinal, principalmente no íleo e no cólon^3^. A amidação é crucial para o GLP1, pois afeta as funções metabólicas e endócrinas, incluindo a regulação da glicose, lipídios e esvaziamento gástrico^4^. Na década de 1980, durante estudos sobre os genes do proglucagon, o GLP1 foi descoberto e caracterizado. Inicialmente considerado inativo, pesquisas adicionais revelaram que a amidação pós-traducional era essencial para sua ativação. Determinou-se que as formas ativas e funcionais no corpo são GLP1(7-37) e GLP1(7-36)-amida, detectáveis na circulação periférica^2,5^.

## Efeitos Fisiológicos do GLP1

O GLP1 desempenha uma variedade de funções fisiológicas que se concentram na homeostase da glicose, dependendo do estado glicêmico e nutricional dos pacientes^6,7^. Em condições de jejum, a concentração média de GLP1(7-36)--amida é de 7,6 ± 1 pM. No entanto, 90 minutos após a ingestão de alimentos, há um aumento significativo, chegando a 41,6 ± 5 pM. Em contrapartida, o GLP1(7-37) apresenta um aumento mais modesto após a alimentação, com concentrações de 6,6 ± 1 pM em jejum e 10 ± 1 pM após 90 minutos da ingestão alimentar^5^.

O receptor de GLP1 (GLP1R) é composto por 463 aminoácidos e inclui oito domínios hidrofóbicos. A região-chave do peptídeo GLP1(7-36)-amida responsável por sua ligação ao receptor, está localizada nos aminoácidos 7 a 21. Notavelmente, o aminoácido na posição 7 (histidina) na extremidade N-terminal do GLP1 apresenta uma cadeia lateral de imidazol e um grupo amino livre. A cadeia lateral de imidazol é crucial para a interação com o GLP1R, enquanto o grupo amino livre é o local onde a enzima dipeptidil peptidase-4 (DPP-4) realiza a inativação enzimática^2,8,9,10^.

O GLP1 exerce seus efeitos por meio da ativação de receptores pertencentes à família dos receptores metabotrópicos acoplados à proteína G, que estão distribuídos em vários órgãos, como pâncreas, pulmões, coração, rins, estômago, intestino, glândula pituitária, nervo vago e diversas regiões do sistema nervoso central^11^.

O GLP1 atua no pâncreas com o objetivo de reduzir os níveis de glicose no sangue. Isso é alcançado por meio do aumento da síntese e da liberação de insulina, além de estimular a proliferação e inibir a apoptose das células beta, conforme demonstrado em estudos *in vitro* e em animais^7,11,12,13^. É interessante observar que esse aumento na secreção de insulina é proporcional aos níveis de glicose no sangue, e esse é um dos fatores que provavelmente contribui de maneira significativa para a baixa incidência de hipoglicemia com o uso de GLP1-RAs.

## Farmacocinética e Farmacodinâmica dos Agonistas do Receptor de GLP1

Os GLP1-RAs são um grupo de medicamentos que atuam como agentes hipoglicemiantes ao mimetizar a ação do peptídeo GLP1 endógeno. Eles ativam o GLP1R, desencadeando a secreção de insulina dependente de glicose, inibindo a liberação de glucagon e retardando o esvaziamento gástrico. Além disso, esses medicamentos possuem uma meia-vida mais longa do que os peptídeos endógenos porque sua estrutura os torna resistentes à inativação pela enzima DPP-4^13^.

Atualmente, existem seis medicamentos aprovados nessa categoria: exenatida, liraglutida, semaglutida, albiglutida, lixisenatida e dulaglutida. No entanto, a albiglutida não está disponível comercialmente. Cada um desses medicamentos reduz os níveis de hemoglobina glicada em aproximadamente 1%, com um impacto maior em pacientes com pior controle glicêmico^14^.

Além das diferenças estruturais entre os agonistas do GLP1 e o peptídeo GLP1 endógeno, os GLP1-RAs apresentam diferenças estruturais entre si, o que lhes confere características farmacocinéticas e farmacodinâmicas exclusivas. De acordo com sua estrutura, eles podem ser divididos entre os de origem humana, os sintetizados a partir de modificações de fragmentos ativos do GLP1 fisiológico e os gerados pela replicação da estrutura da exendina-4, um peptídeo natural de 39 aminoácidos naturalmente resistente à ação da enzima DPP-4^15^. A classificação segundo as propriedades farmacocinéticas divide esses medicamentos em dois grupos distintos: agonistas de curta duração de ação (exenatida e lixisenatida), com meia-vida de aproximadamente 2 a 3 horas, e agonistas de longa duração de ação (dulaglutida, liraglutida, semaglutida, albiglutida e exenatida de longa duração), com meia-vida de 7 a 13 dias^16^.

## Efeitos Renais dos GLP1-RAs

O GLP1R foi identificado em diferentes partes da unidade tubuloglomerular. Estudos em humanos identificaram sua expressão em células tubulares proximais e em células musculares lisas vasculares pré-glomerulares^17^. Já em modelos animais, diversos estudos localizaram o GLP1R em células glomerulares e na porção proximal do túbulo contorcido proximal^18,19^.

Além disso, os GLP1-RAs parecem induzir um aumento na excreção de sódio e no volume urinário por meio de ação direta no túbulo proximal. Esse efeito, que foi dose-dependente, também foi associado a uma redução na TFG, sugerindo um potencial efeito renoprotetor dos GLP1-RAs desde meados da década de 2000^20,21^.

A maior parte das evidências sobre os impactos hemodinâmicos no nível glomerular vem de estudos com animais e modelos *in vitro*, o que limita, em certa medida, a extrapolação desses achados para condições fisiológicas em humanos^22^.

Alguns modelos animais forneceram evidências que sugerem que o GLP1 é um fator natriurético fisiologicamente relevante que contribui para o equilíbrio de sódio, em parte pela modulação tonal da atividade da isoforma 3 do trocador de sódio-hidrogênio (NHE3) na borda em escova do túbulo contorcido proximal^23,24^. A natriurese induzida por esses hormônios ativa o feedback tubuloglomerular e, como resultado, promove a vasoconstrição reflexa da arteríola aferente glomerular^19^.

Por outro lado, o papel vascular dos GLP1-RAs parece ser explicado por sua ação na endotelina-1, um potente vasoconstritor que pode contribuir para a disfunção renal e progressão da doença clínica^25^. Estudos *in vitro* e *in vivo* demonstraram a ação dos GLP1-RAs através da supressão da endotelina-1 em nível vascular, ao reduzir a fosforilação do fator nuclear kappa B (NF-κB) na presença de concentrações elevadas de glicose, como ocorre em pacientes com diabetes mellitus tipo 2 (DM2). Isso reduz a vasoconstrição e melhora o fluxo sanguíneo renal^26^.

Além disso, a ativação do feedback tubuloglomerular reduz a secreção de renina através de um sinal inibitório no aparelho justaglomerular, induzindo reflexivamente a vasodilatação da arteríola eferente glomerular. Dessa forma, o GLP1 faz parte dos mecanismos regulatórios envolvidos na homeostase do sistema renina-angiotensina-aldosterona, o qual geralmente está comprometido em pacientes com nefropatia^27,28^. Pesquisas adicionais relataram que o GLP1 pode regular negativamente a expressão tecidual de angiotensina II, o que pode levar a processos fisiológicos benéficos, reduzindo a hipertensão glomerular, a secreção de aldosterona e o tônus das arteríolas eferentes renais^28^. Dessa forma, são evidenciados diversos mecanismos antagonistas que, paradoxalmente, modificam a hemodinâmica renal; no entanto, há também outros mecanismos que inclinam o equilíbrio no sentido de um efeito nefroprotetor líquido em pacientes que utilizam análogos desses medicamentos. A Figura 1 resume os efeitos renais glomerulares e tubulares conhecidos do GLP1.

**Figura 1:** Efeito do GLP1 no glomérulo e nos túbulos renais: A estimulação do receptor de GLP1 induz a vasodilatação da arteríola aferente mediada por um aumento na disponibilidade de óxido nítrico (vasodilatação mediada por NO). Há também vasodilatação direta na arteríola aferente. Isso resulta em um aumento da pressão intraglomerular. Por outro lado, o GLP1 diminui a resistência vascular na arteríola eferente através de mediadores antagonistas, como a endotelina 1 (ET1) e a angiotensina 2 (ANGII), resultando em vasodilatação pós-glomerular e, consequentemente, em uma redução da pressão intraglomerular. Nos túbulos, o GLP1 induz um efeito natriurético, reduzindo a atividade e a expressão dos cotransportadores de sódio-glicose tipo I e II (SGLT1/2), além de reduzir a atividade do trocador NH3 através da fosforilação, levando ao aumento da presença de Na+ na mácula densa, restaurando o feedback tubuloglomerular com vasoconstrição aferente e redução das pressões intraglomerulares. Há também uma diminuição na liberação de glucagon, que está relacionada à redução da reabsorção de Na+ no túbulo contorcido proximal. Em resumo, o efeito líquido sobre a taxa de filtração glomerular irá depender da magnitude do efeito dominante nos mecanismos com efeitos opostos, da taxa de filtração glomerular basal e das características individuais de cada paciente. GLP1: Peptídeo-1 semelhante ao glucagon.

Dentro desses mecanismos adicionais, pesquisas relacionadas revelaram que os GLP1-RAs reduzem a expressão dos receptores AT1, favorecendo a função dos receptores AT2 nos capilares glomerulares e nos túbulos proximais do néfron. Esse efeito contribui para a redução da fibrose induzida pela angiotensina 2^28^. Por outro lado, alguns modelos animais estabeleceram que a administração de GLP1-RAs aumenta a atividade da enzima conversora de angiotensina em diferentes órgãos, particularmente no rim, cuja atividade enzimática é comprometida em cenários de insuficiência renal^29^.

Outro efeito nefroprotetor potencial do GLP1 está relacionado à sua função antioxidante, que é realizada através da regulação negativa do fator nuclear-kB mediada por superóxido e da supressão da enzima NADPH oxidase 4 (NOX-4)^30,31^. Esta enzima é induzível por meio de uma cascata de transdução dependente de angiotensina 2. A NOX-4 promove a produção de espécies reativas de oxigênio, levando à hiperativação do sistema renina-angiotensina-aldosterona, bem como à disfunção endotelial e à nefropatia diabética. Além de inibir a produção tecidual de angiotensina 2, o GLP1 ativa a via de transdução dependente de adenosina monofosfato cíclico (AMPc) e a proteína quinase A, que contrabalança negativamente a expressão de NOX-4 e reduz os efeitos derivados do estresse oxidativo^32,33^. A Figura 2 descreve os mecanismos anti-inflamatórios e a redução do estresse oxidativo induzidos pelo GLP1.

**Figura 2:** Efeito do estresse anti-inflamatório e antioxidante do GLP1: a estimulação do receptor de GLP1 ativa vias de sinalização intracelular, como a proteína quinase A, levando a um aumento subsequente na concentração de mensageiros secundários, como a adenosina monofosfato cíclico (AMPc), que exerce um efeito inibitório na nicotinamida adenina dinucleotídeo fosfato (NADPH) oxidase, uma enzima importante na produção de espécies reativas de oxigênio (ERO). Além disso, há uma regulação positiva da heme oxigenase 1 (HO1), que inibe a produção de EROs. O aumento nas concentrações de AMPc regula negativamente a sinalização da apoptose e a atividade do fator nuclear Kappa Beta (NF-κB), resultando em uma redução subsequente na produção de citocinas e quimiocinas pró-inflamatórias. DPP4: Dipeptidil peptidase-4, GLP1: Peptídeo-1 semelhante ao glucagon.

O efeito antialbuminúrico relacionado às funções fisiológicas do GLP1 tem sido relatado como um fenômeno subjacente associado à diminuição da atividade plasmática da renina, à indução de natriurese e à redução do estresse oxidativo^21^.

## Desfechos Renais

Com as mudanças regulatórias instituídas pela FDA no final dos anos 2000, estudos de segurança cardiovascular de grande porte tornaram-se obrigatórios para novos medicamentos destinados ao tratamento do diabetes. Esse requisito proporcionou a oportunidade de explorar desfechos secundários relevantes, incluindo aqueles relacionados à doença renal, que são de particular interesse para nós.

Nos últimos anos, diversos estudos sobre diferentes agonistas do receptor GLP1 (GLP1-RAs) mostraram dados promissores em relação a essa nova classe de medicamentos para o tratamento da progressão da doença renal crônica (DRC). O estudo LEADER^34^ constatou que a administração de liraglutida em pacientes diabéticos foi associada a uma redução significativa na incidência de nefropatia como um evento microvascular em comparação com o grupo placebo (HR 0,78; IC 95% 0,67 - 0,92; p=0,003). Além disso, o uso de liraglutida também foi associado a uma menor incidência de doença renal crônica, evidenciando uma preservação significativa no número de eventos com taxa de filtração glomerular (TFG) entre 30 e 59 mL/min/1,73 m^2^.

De maneira semelhante, dados do estudo SUSTAIN-6^35^, que comparou a administração de semaglutida com placebo, revelaram uma notável função nefroprotetora dos GLP1-RAs. Isso foi associado a uma redução significativa na incidência de macroalbuminúria e a um número menor de eventos de redução da TFG. Além disso, o estudo REWIND^36^ revelou diferenças significativas que favorecem o uso de dulaglutida comparado ao placebo em relação à macroalbuminúria *de novo* e à redução da função renal com TFG > 30 mL/min/1,73 m^2^. O estudo HARMONY relatou que, em pacientes tratados com albiglutida, há uma incidência menor de insuficiência renal com TFG ≥60 to <90 mL/min/1,73 m^2^ em comparação com o grupo placebo^37^. No estudo AMPLITUDE-O^38^, constatou-se uma incidência menor de macroalbuminúria e deterioração na função renal em pacientes diabéticos tratados com efpeglenatida em comparação com aqueles que receberam placebo. Isso ressalta a necessidade de mais evidências para confirmar se esses medicamentos são verdadeiramente nefroprotetores em pacientes com DRC associada ao diabetes tipo 2. Estudos de mundo real confirmaram os mesmos resultados encontrados em ensaios controlados, demonstrando a eficácia, a segurança e a redução da albuminúria com os GLP1-RAs^39,40^.

Considerando todos esses resultados promissores, é absolutamente natural projetar e conduzir um estudo com os GLP1-RAs com foco principalmente nos desfechos renais. O estudo FLOW com a semaglutida foi o primeiro estudo especificamente desenhado para confirmar os benefícios renais desse medicamento em uma população de pacientes diabéticos com risco cardiovascular muito elevado^41^. O uso de 1 mg de semaglutida resultou em uma redução de 24% no desfecho composto primário, que incluiu uma redução persistente na TFGe de mais de 50%, atingindo uma TFGe inferior a 15 mL/min/1,73 m^2^, início de terapia renal substitutiva e morte renal ou cardiovascular. Todos os resultados dos outros desfechos pré-especificados na análise hierárquica também foram favoráveis à semaglutida, incluindo a comparação das inclinações das curvas da taxa de filtração glomerular (TFG), eventos cardiovasculares adversos maiores (ECAM) e até mesmo mortalidade por todas as causas.

A publicação do estudo FLOW, que ocorreu não mais do que duas semanas antes da conclusão desta revisão, ainda não forneceu uma análise de mediação. Essa análise ajudaria a determinar quanto do efeito observado foi devido a efeitos indiretos, como perda de peso e redução de HbA1c, em oposição a um possível efeito direto da semaglutida. O último pode incluir um melhor controle do estresse oxidativo, uma ação anti-inflamatória, indução de natriurese e redução da hipertensão glomerular. A Figura 3 resume os resultados individuais de todos esses estudos para uma melhor visualização do impacto dos GLP1-RAs nos desfechos renais.

**Figura 3:** Desfechos renais compostos em ensaios clínicos com GLP1.

## Uso de Análogos de GLP1 em Pacientes Obesos não Diabéticos

Devido aos bons resultados com os GLP1-RAs em pacientes diabéticos, era de se esperar que o estudo dos efeitos desses medicamentos também começasse a ser explorado em pacientes não diabéticos. Trata-se de algo semelhante ao que observamos recentemente em nefrologia com os inibidores do SGLT2 e que está atualmente em andamento com a finerenona.

Dados de mais de quarenta ensaios clínicos realizados em pacientes obesos sem diabetes indicam um efeito nefroprotetor absoluto^42,43,44^. No ensaio STEP 3, observou-se uma porcentagem significativamente maior de pacientes com função renal normal (TFGe ≥90 mL/min/1,73 m^2^) no grupo tratado com semaglutida em comparação ao grupo placebo (68,8% *vs.* 65,2%)^45^. Por outro lado, em um ensaio clínico randomizado duplo-cego, não foram observados casos de deterioração ou insuficiência renal em pacientes tratados com exenatida e dapagliflozina. No entanto, é importante observar que esses eventos ocorreram em todos os braços do estudo, destacando a necessidade de monitoramento contínuo^46^. Recentemente, o estudo TRIAL demonstrou uma incidência menor de óbito por causas renais, início de terapia renal substitutiva de longo prazo (diálise ou transplante), TFGe persistente abaixo de 15 mL por minuto por 1,73 m^2^, redução persistente de 50% na TFGe em comparação com o valor basal e início de macroalbuminúria persistente em pacientes tratados com semaglutida em comparação com placebo^47^. Além disso, no estudo exploratório de Heerspink et al^48^, foram analisados os dados dos ensaios STEP 1, STEP 2 e STEP 3, onde se constatou que a relação albumina/creatinina urinária melhorou em uma proporção maior de pacientes que receberam semaglutida 1,0 mg e 2,4 mg em comparação com o placebo. Também é importante destacar que foi observada uma redução significativa da albuminúria na população não diabética envolvida nesses estudos. Isso sugere a possibilidade de efeitos nefroprotetores diretos do medicamento neste grupo, além do efeito indireto de redução de peso.

Por fim, publicamos recentemente os resultados do estudo SELECT sobre desfechos renais. Utilizando dados de mais de 16.000 pacientes, metade dos quais recebeu semaglutida na dose de 2,4 mg por semana, os autores relataram uma melhora nos desfechos renais mesmo em pacientes sem diabetes. O desfecho composto do estudo SELECT difere daquele do estudo FLOW por incluir o início de macroalbuminúria persistente como um de seus componentes. A redução observada no desfecho composto foi muito semelhante à do estudo FLOW, com uma redução de 22% ao longo de 104 semanas de acompanhamento^49^.

## Conclusões

A cada dia, aprendemos mais sobre os efeitos renais dos GLP1-RAs. Esses mecanismos de ação no sistema renal podem ser classificados em diretos e indiretos. No entanto, é preciso continuar aprofundando a compreensão das vias moleculares por meio das quais os GLP1-RAs exercem seu impacto na função renal, uma vez que os dados clínicos sugerem que essa classe terapêutica de medicamentos pode ter efeitos nefroprotetores significativos. A maior parte da literatura que fornece dados clínicos sobre desfechos renais é derivada de estudos fundamentais realizados em pacientes diabéticos, com ou sem obesidade. Portanto, devemos aprimorar as evidências em pacientes obesos sem diabetes e realizar estudos específicos em populações com doença renal crônica já definida.
